# 4-Methyl-2*H*-1,3-oxazine-2,6(3*H*)-dione

**DOI:** 10.1107/S1600536809034618

**Published:** 2009-09-05

**Authors:** Damon Parrish, Fredrick Leuschner, Gretchen M. Rehberg, Margaret E. Kastner

**Affiliations:** a Department of Chemistry, Bucknell University, Lewisburg, PA 17837, USA

## Abstract

In the title compound, C_5_H_5_NO_3_, the planar (maximum deviation = 0.075 Å for the ring O atom) mol­ecules form N—H⋯O hydrogen bonds in a zigzag chain (C—O⋯N bond angle ≃ 140°) between glide-related mol­ecules.

## Related literature

For synthetic background, see: Warren *et al.* (1975 ▶); Rehberg & Glass (1995 ▶). For related structures, see: Copley *et al.* (2005 ▶); Parrish, Leuschner *et al.* (2009 ▶); Parrish, Tivitmahaisoon *et al.* 2009 ▶).
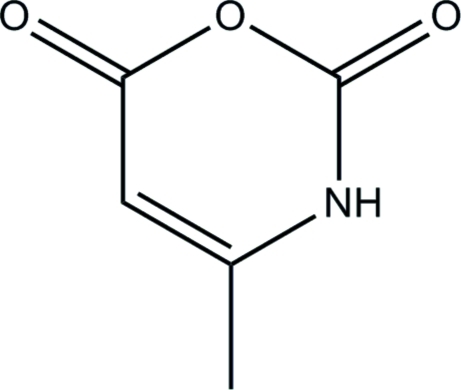


## Experimental

### 

#### Crystal data

C_5_H_5_NO_3_
*M* *_r_* = 127.1
Monoclinic, 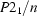

*a* = 7.254 (3) Å
*b* = 6.683 (2) Å
*c* = 11.689 (5) Å
β = 98.11 (4)°
*V* = 561.0 (4) Å^3^
*Z* = 4
Mo *K*α radiation
μ = 0.13 mm^−1^
*T* = 293 K
0.46 × 0.30 × 0.10 mm

#### Data collection

Bruker R3/V diffractometer
Absorption correction: none
1410 measured reflections
1294 independent reflections
910 reflections with *I* > 2σ(*I*)
*R*_int_ = 0.012
3 standard reflections every 97 reflections intensity decay: none

#### Refinement

*R*[*F* ^2^ > 2σ(*F* ^2^)] = 0.049
*wR*(*F* ^2^) = 0.191
*S* = 0.93
1294 reflections
83 parameters
H-atom parameters constrained
Δρ_max_ = 0.23 e Å^−3^
Δρ_min_ = −0.24 e Å^−3^

### 

Data collection: *XSCANS* (Bruker, 1996 ▶); cell refinement: *XSCANS* (Bruker, 1996 ▶); data reduction: *XSCANS* (Bruker, 1996 ▶); program(s) used to solve structure: *SHELXS97* (Sheldrick, 2008 ▶); program(s) used to refine structure: *SHELXL97* (Sheldrick, 2008 ▶); molecular graphics: *SHELXTL* (Sheldrick, 2008 ▶); software used to prepare material for publication: *SHELXTL* (Sheldrick, 2008 ▶).

## Supplementary Material

Crystal structure: contains datablocks I, global. DOI: 10.1107/S1600536809034618/pv2197sup1.cif

Structure factors: contains datablocks I. DOI: 10.1107/S1600536809034618/pv2197Isup2.hkl

Additional supplementary materials:  crystallographic information; 3D view; checkCIF report

## Figures and Tables

**Table 1:** Hydrogen-bond geometry (Å, °)

*D*—H⋯*A*	*D*—H	H⋯*A*	*D*⋯*A*	*D*—H⋯*A*
N3—H3⋯O6^i^	0.86	2.02	2.877 (3)	173

Symmetry code: (i) 
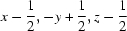
.

**Fig. 1.**
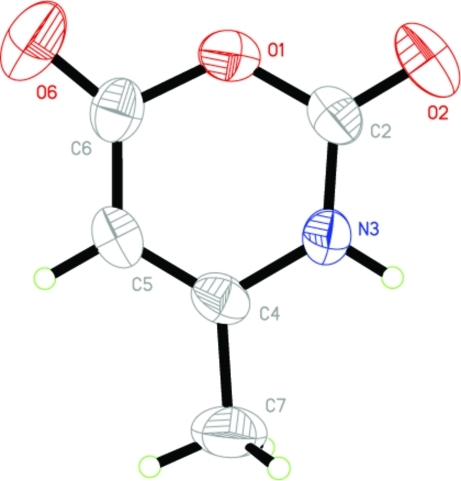
The molecular structure of the title compound, with atom labels and 50% probability displacement ellipsoids for non-H atoms.

**Fig. 2.**
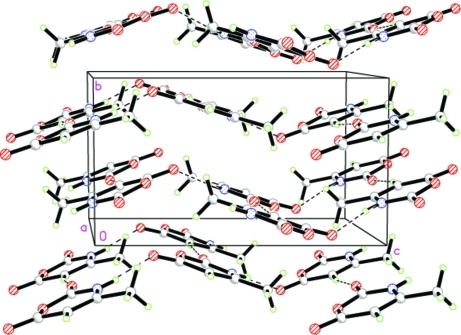
The packing of the title compound viewed down the a axis; intermolecular hydrogen bonds have been represented by dashed lines.

## supplementary crystallographic information

### Comment

The synthesis of derivatives of 3-oxauracil has previously been reported (Warren *et al.*, 1975) and an improved synthesis of the unsubstituted 3-oxauracil was reported by Rehberg & Glass (1995). The structure of the unsubstituted 3-oxauracil and its monohydrate have been reported (Copley *et al.*, 2005). Three derivatives of 3-oxauracil (4-methyl, 4-bromo, and 4,5-dichloro) have been prepared in our laboratory in route to the synthesis of 1-aza-1,3-butadienes. In this paper, we report the crystal structure of the title compound, (I).

In the title compound (Fig. 1) only one intermolecular H-bond is formed between N3 and O6 of glide-related molecules (details are given in Table 1). Although the molecules of (I) are planar, the H-bonding chains are staggered as shown in Figure 2. The hydrogen bonding networks in (I) differs significantly from the hydrogen bonding in 4,5-dichloro (Parrish, Leuschner *et al.*, 2009) and 4-bromo (Parrish, Tivitmahaisoon *et al.*, 2009) derivatives.

### Experimental

Citraconic anhydride (3-methylfuran-2,5-dione, 2.0 ml, 22 mmol) and trimethylsilyl azide (3.0 ml, 23 mmol) were added to 10 ml dichloromethane at 273 K and stirred under nitrogen for 4 h. Upon warming to room temperature over night, a white precipitate formed. Ethanol (2.5 ml) was added, the mixture stirred 2 additional hours, and then the solvent was removed under reduced pressure to obtain the title compound; yield: 1.7 g (13 mmol, 59%). Crystals of the title compound were grown from a solution of acetone at room temperature by slow evaporation.

### Refinement

Hydrogen positions were calculated and refined using a riding model using the following C—H distances: methyl 0.96 Å, methylene 0.93 Å, and N—H 0.88 Å with *U*_iso_(H) = 1.2*U*_eq_(C5/N3) and 1.5*U*_eq_(C7).

### Crystal data

**Table:**

C_5_H_5_NO_3_	*F*(000) = 264
*M**_r_* = 127.1	*D*_x_ = 1.505 Mg m^−^^3^*D*_m_ = 1.46 Mg m^−^^3^*D*_m_ measured by floatation in bromoform/hexane solution
Monoclinic, *P*2_1_/*n*	Mo *K*α radiation, λ = 0.71073 Å
Hall symbol: -P 2yn	Cell parameters from 20 reflections
*a* = 7.254 (3) Å	θ = 10–12.5°
*b* = 6.683 (2) Å	µ = 0.13 mm^−^^1^
*c* = 11.689 (5) Å	*T* = 293 K
β = 98.11 (4)°	Plates, colorless
*V* = 561.0 (4) Å^3^	0.46 × 0.30 × 0.10 mm
*Z* = 4	

### Data collection

**Table:**

Bruker R3/V diffractometer	*R*_int_ = 0.012
Radiation source: fine-focus sealed tube	θ_max_ = 27.6°, θ_min_ = 3.1°
graphite	*h* = 0→9
θ – 2θ scans	*k* = 0→8
1410 measured reflections	*l* = −15→15
1294 independent reflections	3 standard reflections every 97 reflections
910 reflections with *I* > 2σ(*I*)	intensity decay: none

### Refinement

**Table:**

Refinement on *F*^2^	Primary atom site location: structure-invariant direct methods
Least-squares matrix: full	Secondary atom site location: difference Fourier map
*R*[*F*^2^ > 2σ(*F*^2^)] = 0.049	Hydrogen site location: inferred from neighbouring sites
*wR*(*F*^2^) = 0.191	H-atom parameters constrained
*S* = 0.93	*w* = 1/[σ^2^(*F*_o_^2^) + (0.1301*P*)^2^ + 0.1905*P*] where *P* = (*F*_o_^2^ + 2*F*_c_^2^)/3
1294 reflections	(Δ/σ)_max_ = 0.005
83 parameters	Δρ_max_ = 0.23 e Å^−^^3^
0 restraints	Δρ_min_ = −0.24 e Å^−^^3^

### Special details

**Table:**

Geometry. All e.s.d.'s (except the e.s.d. in the dihedral angle between two l.s. planes) are estimated using the full covariance matrix. The cell e.s.d.'s are taken into account individually in the estimation of e.s.d.'s in distances, angles and torsion angles; correlations between e.s.d.'s in cell parameters are only used when they are defined by crystal symmetry. An approximate (isotropic) treatment of cell e.s.d.'s is used for estimating e.s.d.'s involving l.s. planes.
Refinement. Refinement of *F*^2^ against ALL reflections. The weighted *R*-factor *wR* and goodness of fit *S* are based on *F*^2^, conventional *R*-factors *R* are based on *F*, with *F* set to zero for negative *F*^2^. The threshold expression of *F*^2^ > σ(*F*^2^) is used only for calculating *R*-factors(gt) *etc*. and is not relevant to the choice of reflections for refinement. *R*-factors based on *F*^2^ are statistically about twice as large as those based on *F*, and *R*- factors based on ALL data will be even larger.

### Fractional atomic coordinates and isotropic or equivalent isotropic displacement parameters (Å^2^)

**Table:**

	*x*	*y*	*z*	*U*_iso_*/*U*_eq_	
O1	0.6042 (2)	0.1383 (2)	0.68042 (13)	0.0466 (5)	
C2	0.4998 (3)	0.2078 (3)	0.58306 (19)	0.0422 (5)	
O2	0.3337 (2)	0.2161 (3)	0.57888 (19)	0.0694 (6)	
N3	0.5939 (2)	0.2571 (3)	0.49490 (14)	0.0406 (5)	
H3	0.5305	0.2967	0.4312	0.049*	
C4	0.7833 (3)	0.2477 (3)	0.50089 (18)	0.0392 (5)	
C5	0.8847 (3)	0.1896 (3)	0.59971 (19)	0.0430 (5)	
H5	1.0137	0.1846	0.6048	0.052*	
C6	0.7988 (3)	0.1357 (3)	0.69668 (18)	0.0435 (5)	
O6	0.8682 (3)	0.0868 (3)	0.79242 (15)	0.0695 (7)	
C7	0.8619 (4)	0.3002 (5)	0.3938 (2)	0.0630 (8)	
H7A	0.9955	0.2979	0.4093	0.095*	
H7B	0.8210	0.4317	0.3687	0.095*	
H7C	0.8201	0.2050	0.3343	0.095*	

### Atomic displacement parameters (Å^2^)

**Table:**

	*U*^11^	*U*^22^	*U*^33^	*U*^12^	*U*^13^	*U*^23^
O1	0.0448 (9)	0.0555 (10)	0.0417 (8)	−0.0028 (7)	0.0137 (6)	0.0020 (7)
C2	0.0329 (10)	0.0455 (12)	0.0490 (11)	0.0003 (8)	0.0086 (8)	−0.0068 (9)
O2	0.0324 (9)	0.0847 (14)	0.0936 (15)	−0.0020 (9)	0.0174 (9)	−0.0026 (11)
N3	0.0342 (9)	0.0511 (10)	0.0350 (9)	0.0041 (7)	0.0003 (7)	0.0010 (7)
C4	0.0374 (10)	0.0384 (10)	0.0445 (11)	0.0029 (8)	0.0152 (8)	−0.0025 (9)
C5	0.0294 (9)	0.0458 (12)	0.0534 (12)	0.0012 (9)	0.0049 (8)	−0.0021 (10)
C6	0.0447 (11)	0.0395 (11)	0.0435 (11)	−0.0024 (9)	−0.0038 (9)	−0.0025 (9)
O6	0.0831 (14)	0.0687 (13)	0.0494 (10)	−0.0096 (10)	−0.0167 (9)	0.0110 (9)
C7	0.0651 (16)	0.0733 (18)	0.0573 (14)	0.0053 (13)	0.0313 (12)	0.0133 (13)

### Geometric parameters (Å, °)

**Table:**

O1—C2	1.357 (3)	C4—C7	1.489 (3)
O1—C6	1.398 (3)	C5—C6	1.415 (3)
C2—O2	1.200 (3)	C5—H5	0.9300
C2—N3	1.354 (3)	C6—O6	1.206 (3)
N3—C4	1.368 (3)	C7—H7A	0.9600
N3—H3	0.8600	C7—H7B	0.9600
C4—C5	1.337 (3)	C7—H7C	0.9600
			
C2—O1—C6	123.50 (17)	C4—C5—H5	119.5
O2—C2—N3	124.6 (2)	C6—C5—H5	119.5
O2—C2—O1	119.2 (2)	O6—C6—O1	114.3 (2)
N3—C2—O1	116.10 (18)	O6—C6—C5	129.7 (2)
C2—N3—C4	124.08 (18)	O1—C6—C5	115.99 (18)
C2—N3—H3	118.0	C4—C7—H7A	109.5
C4—N3—H3	118.0	C4—C7—H7B	109.5
C5—C4—N3	118.95 (18)	H7A—C7—H7B	109.5
C5—C4—C7	124.5 (2)	C4—C7—H7C	109.5
N3—C4—C7	116.6 (2)	H7A—C7—H7C	109.5
C4—C5—C6	121.03 (19)	H7B—C7—H7C	109.5
			
C6—O1—C2—O2	−175.5 (2)	N3—C4—C5—C6	0.9 (3)
C6—O1—C2—N3	6.7 (3)	C7—C4—C5—C6	−177.8 (2)
O2—C2—N3—C4	179.7 (2)	C2—O1—C6—O6	173.2 (2)
O1—C2—N3—C4	−2.6 (3)	C2—O1—C6—C5	−6.9 (3)
C2—N3—C4—C5	−1.1 (3)	C4—C5—C6—O6	−177.3 (2)
C2—N3—C4—C7	177.7 (2)	C4—C5—C6—O1	2.8 (3)

### Hydrogen-bond geometry (Å, °)

**Table:**

*D*—H···*A*	*D*—H	H···*A*	*D*···*A*	*D*—H···*A*
N3—H3···O6^i^	0.86	2.02	2.877 (3)	173

Symmetry codes: (i) *x*−1/2, −*y*+1/2, *z*−1/2.
